# Stability of Blood DNA Methylation Across Two Timepoints in Three Cohorts

**DOI:** 10.3390/biomedicines12112557

**Published:** 2024-11-08

**Authors:** Mikołaj Danielewski, Jarosław Walkowiak, Karolina Wielgus, Jan Krzysztof Nowak

**Affiliations:** Department of Pediatric Gastroenterology and Metabolic Diseases, Poznan University of Medical Sciences, ul. Szpitalna 27/33, 60-572 Poznan, Poland; mikolaj.danielewski@student.ump.edu.pl (M.D.); jarwalk@ump.edu.pl (J.W.); kwielgus@ump.edu.pl (K.W.)

**Keywords:** methylation, methylome, epigenetics, hyperstable, invariant, reliability, tissue, whole blood, methylation array

## Abstract

**Background:** DNA methylation mediates the gene–environment interactions, with implications for health and disease. Studies with sampling at more than one timepoint revealed the considerable variability of the blood methylome, but comprehensive resources on genome-wide methylation stability are still lacking. We aimed to identify methylation sites that remain the most stable across two timepoints in human whole blood. **Methods:** Publicly available blood DNA methylation data from three cohorts were analysed, which included methylation profiles at two timepoints >1 year apart. The cohorts included pre-/post-pubertal children (Illumina 450k array), the elderly (Illumina 450k array), and middle-aged adults with obesity (Illumina EPIC array). Two metrics were used for the stability assessment: the mean absolute difference (MAD) of beta values between two measurements and the intraclass correlation coefficient (ICC). We searched for probes demonstrating high stability (low MAD and high ICC) across the three cohorts. Data from 51 children, 86 elderly adults, and 120 middle-aged participants were re-analysed. **Results:** The median interquartile range (IQR) of the maximum (from three datasets) MAD was 2.1% (1.5–2.9%), and the median of the minimum ICC agreement coefficient was 0.053 (−0.077–0.304). The Pearson’s correlation coefficient for the ICC vs. maximum MAD was low (r = 0.34, *p* < 2.2 × 10^−16^). We found only 239 probes that were highly stable based on both the maximum MAD (<5th percentile, <0.01) and ICC criterion (>95th percentile, >0.74). **Conclusions:** The whole-blood DNA methylation profile, as measured using microarrays, is dynamic over >1 year, but contains a fraction of stable probes, most of which are related to genomic variation. A resource describing probe stability is made publicly available, with the intention to support biomarker studies and the investigation of early epigenetic programming. The absolute error and correlation are two complementary facets of probe stability that may be considered in further research, especially to determine the stability of probes in health and disease across different tissues and populations.

## 1. Introduction

DNA methylation profiles mediate the interaction between genetic information and the environment [1,2]. This is thought to occur dynamically, and also through early-life epigenetic programming which introduces a layer of genomic control with potentially life-long effects [3]. The analysis of methylation profiles provides important information about health and disease, with much hope for designing diagnostic and prognostication tools to empower personalised medicine. In the broadest terms, this is exemplified by global hypomethylation, which was found to be an early biomarker of cancer in peripheral blood mononuclear cells [4]. However, methylation at sets of individual probes (signatures) is now the focus of research, with potential applications in oncology and drug metabolism [4,5], but also rheumatoid arthritis, lupus, diabetes, metabolic syndrome, psoriatic arthritis, Alzheimer’s disease, and other conditions across virtually all medical specialties [6,7,8,9,10]. Moreover, the progress in understanding the human methylation profiles was especially prominent in the field of inflammatory bowel diseases (IBD). In 2014, Adams et al. [11] demonstrated that the analysis of blood methylation at two sites could be sufficient for the diagnosis of Crohn’s disease in children. Further studies on this topic have been summarised in the meta-analysis by Joustra et al. [12], who showed that consistent methylation signals are strongly associated with an IBD diagnosis.

Recently, a blood biomarker containing a set of four probes, with a high potential of clinical translation, was proposed by Noble et al. [13], with a high discriminatory value, which was retained in patients with low C-reactive protein. Additional work was carried out by Kalla et al. [14], who linked blood DNA methylation at *TAP1* and two other genes to the need for IBD treatment escalation, while Joustra et al. [15] showed the potential of blood DNA methylation profiles (53 methylation probes) to predict the response of IBD to tofacitinib. IBD research exemplifies the great potential that methylation profiling has for clinical medicine, and the fact that sets of individual probes are of growing interest. However, Joustra et al. [15] also pointed towards an important limitation of the work on human blood DNA methylation profiles. They critically characterised the limited reliability of blood methylation measurement between timepoints in patients with IBD. Another of their findings pointed at the dependence of the probe stability on genetic information: loci related to the human leukocyte antigen (HLA) region (known for its complex genotype) had a better intraclass correlation coefficient (ICC) than other sites. Methylation sites under genetic influence were found to be more stable overall.

In another study that was directly related to the problem of the long-term stability of blood DNA methylation, the Stratification and Identification of Immunogenetic and Microbial Markers of Rapid Disease Progression in Children with Crohn’s Disease (RISK) study [16], children with IBD were found to retain few methylation signatures of IBD at follow-up (after the resolution of inflammation), compared with the baseline. It is still unclear if the signals were related to inflammation alone or the presence of some specific immune cell types. Therefore, human blood DNA methylation is known to change, and to remain under genetic influence. Probe stability emerges as a new concept, which is different from reliability. Methylation microarrays were found to contain unreliable and invariant probes [17], and there is growing evidence that methylome (all the methylated and unmethylated cytosines in the genome in a given tissue), as measured using arrays, is unstable over time with only few exceptions [18].

Overall, while analysing varying methylation levels in microarray probes or in differentially methylated regions for ascertaining the phenotype–methylome relationship, an extensively researched subject by the scientific community, the concept of methylation stability is relatively underprioritised. Furthermore, most of the publications focus on researching stability in a specific framework (i.e., a health condition like IBD) or the technical issues of obtaining repeatable results. Notwithstanding these limitations, oncological biomarkers based on methylation have been approved in the USA and China [19].

The progress in epigenomics and IBD research, which motivates our work, leads to several questions. If we are to use blood methylation for diagnostics, do we have the knowledge about the relative stability of signals from methylation probes or the reasons to consider them equal in this respect? Have we defined the metrics to measure the methylation probe stability, which are pertinent for various types of models used for biomarker discovery? Does methylation stability matter for the detection of disease-related signals? Have we identified probes that are stable over time independent of genetic variation, which could be used more easily for deciphering epigenetic programming, proposing new types of normalisation, and potentially enhancing biomarker development? To address some of these uncertainties at least in part, we analysed methylation profiles across two timepoints in three cohorts from different age groups, in healthy individuals, with the hypothesis that this will identify highly stable probes that do not change with time regardless of the age or the length of the period between the first and second sampling.

## 2. Materials and Methods

Three datasets were accessed through BioStudies (ArrayExpress). The first dataset, by Almstrup et al. [20] (E-MTAB-4187), included blood DNA methylation profiles obtained using the Illumina 450k in a group of children in pre- and post-pubertal period. The second dataset, by Tan et al. [21] (E-GEOD-73115), contained blood methylomes of a group of elderly, at the beginning and end of 10-year follow-up, and was also obtained with the Illumina 450k array. The third dataset, by Keller et al. [22] (E-MTAB-8956), comprised blood methylomes of middle-aged, obese participants of a randomised study of dietary interventions (the time between sampling was 18 months), and reported results of the analysis with the Illumina EPIC (850k) arrays. The details of sampling, DNA isolation, bisulfite conversion, and hybridisation for each cohort are given in the respective publications. The order of data presentation is (1) children, (2) elderly, and (3) middle-aged, because the last dataset used the EPIC array and because it included patients with obesity, and is, therefore, not directly comparable, and yet still adequate for discovery of (generalizable) stable probes.

Aforementioned datasets were selected for our analysis because of their relatively large sample size, sampling method (repeated sampling after different time intervals), and differing age ranges between datasets. Utilising datasets from different age groups allows us to speculate that the probes we found are not only stable over analysed periods, but also throughout a majority of person’s life. Including datasets from different age groups is not a limitation, as proving that the stability persists across age groups is an intentional part of the study design.

This study was not interested in finding systematic findings with time or methylation clock-related probes. We have included a group of middle-aged patients with obesity, of whom most were male, and, therefore, we do not describe the results as specifically generalizable in the healthy population. Moreover, the studies were carried out mostly in persons of European descent, and may not be generalizable to African, Middle- or South-American, and Asian populations. We have only included one dataset (albeit relatively the largest), which used the 850k EPIC array. Yet, most probes from the 450k are carried over and the results might be useful for newer studies carried out using the EPIC array, and this is also consistent with the literature [17]. We have not compared the 450k vs. the EPIC array because the study design did not allow for this (different age groups and different time between first and second blood sampling). Whereas Joustra et al. [12,15] used the functional normalisation, we have used the quantile normalisation, which is often proposed for data coming from the same tissue with expected similar global level of methylation. Apart from the reasons listed above, we carried out analyses using beta values, instead of M values, because of their widespread use and more intuitive interpretation. This study did not focus on differences between various age groups in the analysed parameters, and did not consider deconvoluted cell types, the proportions of which may affect blood (leukocyte) DNA methylation profiles. Finally, the Gaphunter (*minfi* version 1.50.0, R version 4.4.1) was used as the frequently used approach for detection of genetic influence, but visual data analysis convinced us of its limited sensitivity among the highly stable probes.

The idat files containing raw data from assessment of microarrays were read with minfi software package and combined using minfi::combineArrays to a format compatible with the Illumina 450k array (stripping additional probes from the EPIC arrays) [23,24,25,26]. Quality check was conducted after functional normalisation of the data (without ratioConvert), and full concordance was confirmed for all datasets between sex reported in the clinical data and sex inferred from methylation characteristics. Of note, functional normalisation was required for automated quality check in minfi pipeline, but is not used for main analyses. Statistical significance (*p* values) was assessed for all probes in all participants from RGChannelSets, separately for each dataset, and probes with *p* values > 0.01 in any of the datasets were excluded. After removal of these probes, there were no samples which would have a mean value greater than 0.01, and, therefore, no samples were excluded. Beta values were obtained using minfi::getBeta function. In this work, beta values are analysed to focus on biological relevance, facilitating calculation of chosen metrics and visualisation. Following the logit transform of the M values to beta values, the data may have increased heteroscedasticity towards the extreme ends and may better expose variability in the mid spectrum of beta values, whereas variability at the fully methylated and unmethylated probes may be relatively less visible. The function sva::ComBat was used for correction of batch-related effects through an empirical Bayes framework. The Illumina microarray plate column and row (where individual sample was assessed) were sequentially corrected for in the dataset by Almstrup et al. [20] and by Tan et al. [21] separately. Thereafter, the combined data were corrected for the dataset (three batches).

After data preparation was accomplished, the mean absolute difference (MAD) was calculated for each probe in each age group (dataset) by subtracting results obtained later (second sample) from results obtained earlier (first sample) and calculating the mean of the absolute values. For each probe, the maximum mean absolute difference (MMAD; the poorest stability of three) was selected from among the three calculated mean absolute differences (MAD, for each dataset) so that probes with low MMAD are consistently stable across the age groups. The Intraclass Correlation Coefficient (ICC) was calculated likewise (irr::icc, type = “agreement”, model = “twoway”; the results were verified against psy::icc) for each probe across two timepoints, in each dataset. The minimum ICC (the poorest stability of three) was selected from three ICCs calculated for the probe in three age groups. Moreover, the paired *t*-test *p*-value and Pearson’s correlation coefficient (almost identical to ICC) were derived similarly. The choice of MMAD and ICC reflects two facets of stability—absolute precision (difference) and preservation of a trend (correlation)—which may be important under different scenarios of use (biomarker/score calculations vs. biologically relevant changes).

Information about the presence of any single-nucleotide polymorphisms (SNPs) close to the probe was taken from the Illumina Human Methylation 450k manifest file (v. 1.2). Of note, data on the probe design, nearest gene, and probe group were also taken from the manifest file. Moreover, minfi::gaphunter [27] was used to find probes with clustering of beta values suggestive of genetic influence. The Gaphunter was applied to data from each age group separately. Probes without nearby SNPs and not identified by the Gaphunter in any of the three age groups were considered unrelated to genetic variation.

We categorised some probes as “hyperstable”, following the naming convention from Joustra et al. [12]. Firstly, probes with ICC > 0.74 (95th percentile) were considered hyperstable by ICC (although we used “agreement” instead of “consistency” for ICC calculation; Joustra et al. used >0.9). Secondly, probes with MMAD <0.01, which corresponded to the 5th percentile, were hyperstable by MMAD. These two facets of methylation stability are further dissected in the discussion. Functional annotation of the most stable probes was carried out using missMethyl::gometh function.

## 3. Results

### 3.1. Basic Characteristics of the Three Cohorts

The number of study participants was as follows: 51 children (20 girls, and 31 boys), 86 elderly (62 females, and 24 males; *n* = 180 samples including duplicates, multiple for few samples), and 120 middle-aged patients with obesity (10 females, and 110 males). The median time between sampling was 6.55 [5.06–6.55] years in children/adolescents, 10 years in the elderly, and 18 months in the middle-aged. The age of children was 9 years [6–11 years] pre-puberty, and 15 years [12–16 years] post-puberty; the age of the middle-aged was 49 years [29–69 years], and the age of the elderly was 76 years [73–82 years].

### 3.2. Detecting Probes Under Influence of Genetic Variance

The final dataset comprised 450,079 methylation probes. The number of probes related to SNPs was 82,491. Gaphunter [27] identified the following number of probes potentially related to genetic variation—i.e., gap probes: 20,171 in children, 40,222 in the elderly, and 14,484 in the middle-aged; overall 54,330 unique probes (some instances overlapped). Of those, 12,151 overlapped with the SNP probes. In total, 124,670 probes are potentially related to genetic variance through SNP or as identified by Gaphunter [27].

### 3.3. Difference in Methylation Levels Across Time

**Figure 1:**
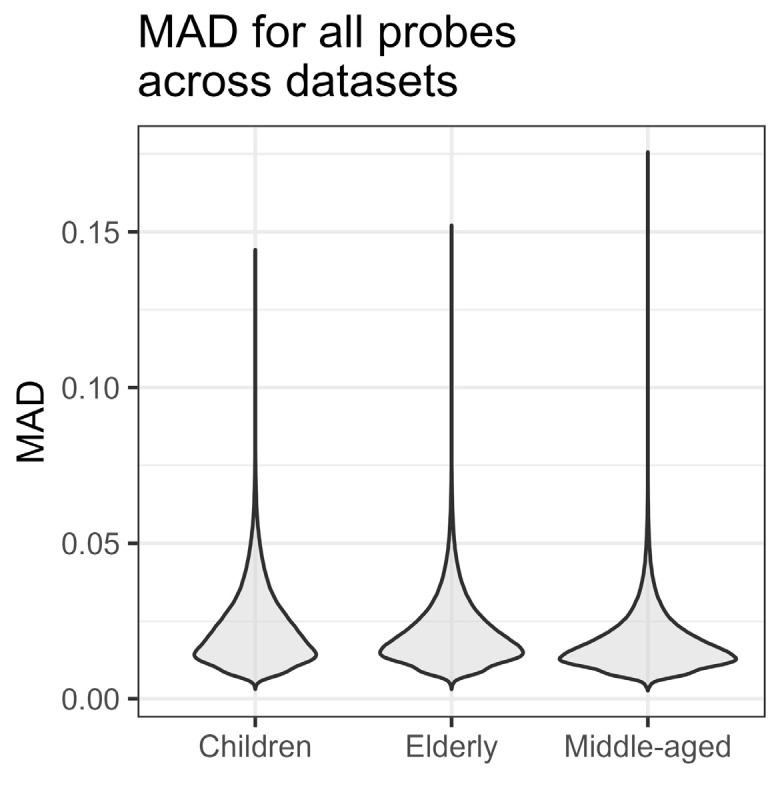
Distribution of mean absolute difference (MAD) across each one of the three datasets.

The median (IQR) of the maximum mean absolute difference (MMAD) was 0.021 (0.015–0.029), with a minimum of 0.003, and a maximum of 0.176. This value was about 20% higher in the gap or SNP probes (0.024 (0.017–0.035) vs. 0.020 (0.014–0.027), *p* < 2.2 × 10^−16^), which does not confirm the greater stability of these probes MMAD-wise, but might be explained by their higher variance. Therefore, from sampling to sampling, the MAD of the beta value was typically only ~2% in absolute terms, which, however, can still be clinically relevant, especially for probes with a low variance. The distribution of the mean absolute difference in datasets is presented in Figure 1.

### 3.4. Stability of Probes: Agreement Score for Methylation Levels Across Time

**Figure 2:**
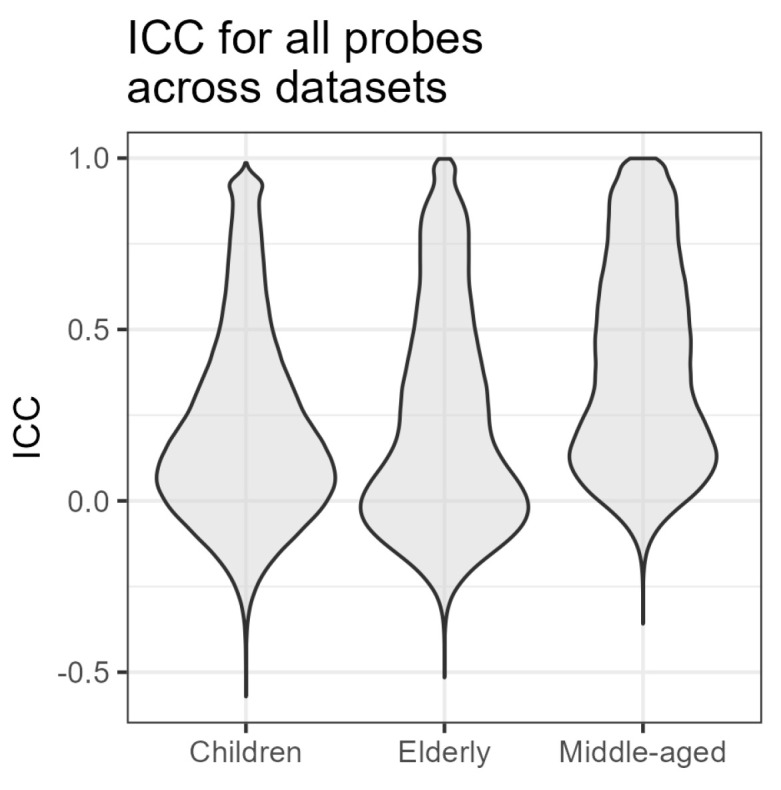
Distribution of intraclass correlation coefficient (ICC) values across the three datasets.

Only a fraction of probes demonstrated a high intraclass correlation coefficient (ICC) between timepoints. The median (IQR) of the minimum ICC agreement coefficient was 0.053 (−0.077–0.304), with a minimum of −0.571, and a maximum of 0.986. The ICC was also higher in probes related to genetics (0.180 (−0.027–0.499) vs. 0.023 (−0.090–0.239), *p* < 2.2 × 10^−16^), which suggested the better stability of these probes. Overall, in most probes, there was no agreement between the two measurements, and a small share of probes showed a good ICC. The distribution of the interclass correlation coefficient between age groups is presented in Figure 2.

### 3.5. Connection Between MMAD and ICC

**Figure 3:**
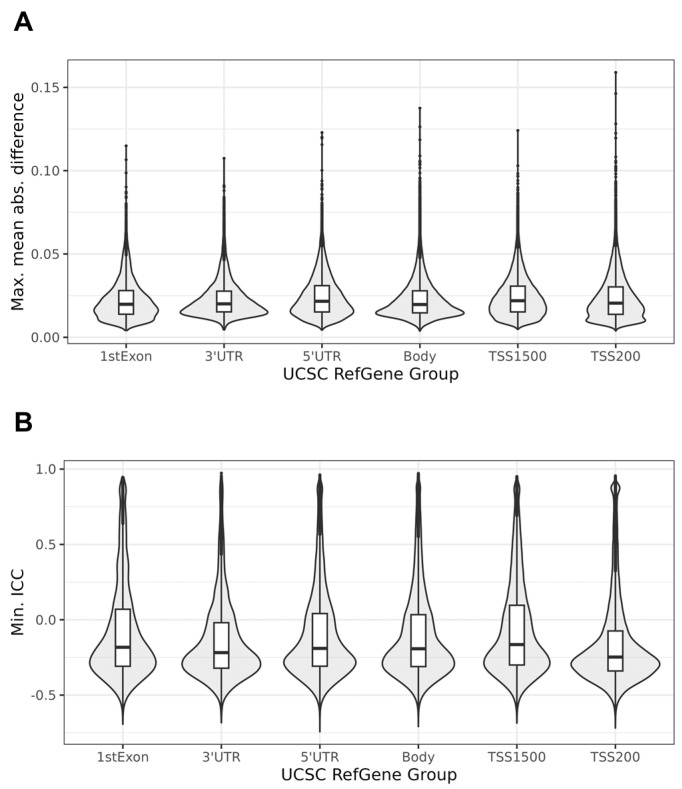
The impact of the location of the probe in a gene on the following: (**A**) maximal mean absolute difference (MMAD), and (**B**) minimal intraclass correlation coefficient (ICC).

**Figure 4:**
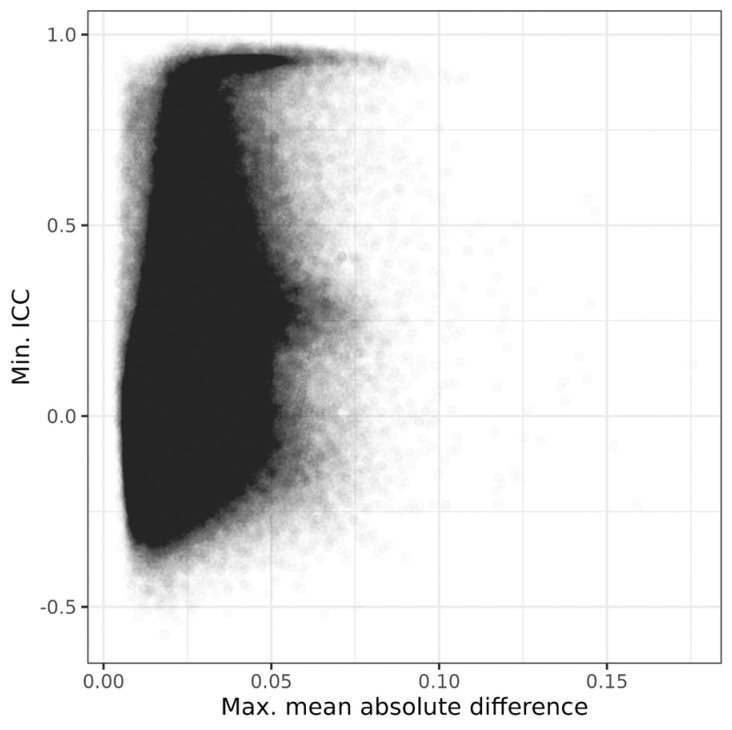
Scatterplot of maximal mean absolute difference (MMAD) vs. minimal intraclass correlation coefficient (ICC), the two measures of stability used to analyse three datasets that, at two separate timepoints, included microarray-based assessments of blood DNA methylome in the same participants.

The relationship between the probe location within a gene and the MMAD or ICC is presented in Figure 3. The relationship between the MMAD and ICC is shown in Figure 4, which demonstrates that the two metrics were, to a large degree, independent. Such independence allowed for a concurrent low MMAD error and high ICC, or vice versa. The Pearson’s correlation coefficient for the ICC vs. MMAD was low, r = 0.340 (*p* < 2.2 × 10^−16^).

**Figure 5:**
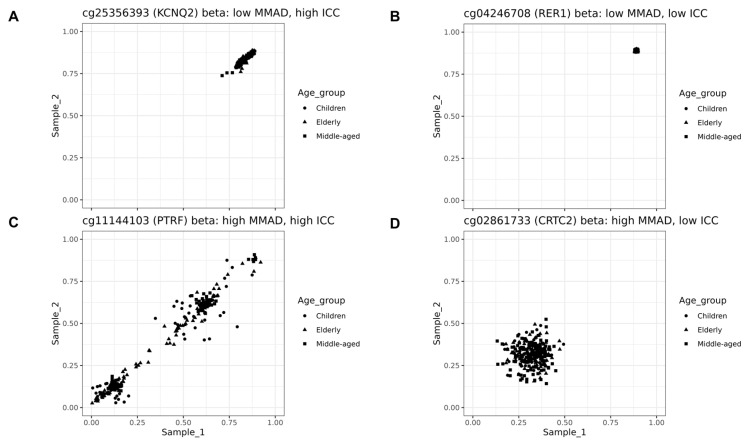
Examples of MMAD-ICC relationships. (**A**) Relationship between methylation levels measured in two points in time demonstrated on the example of probe cg25356393 in gene *KCNQ2*, which had high ICC score and low MMAD value. (**B**) Relationship between methylation levels measured in two points in time demonstrated on the example of probe cg04246708 in *RER1*, which had low value in both MMAD and ICC. (**C**) Relationship between methylation levels measured at two points in time on the example of probe cg11144103 in *PTRF*, which had high MMAD and ICC scores. (**D**) Relationship between methylation levels measured at two points in time for probes with high MMAD values and low ICC score, demonstrated using the example of probe cg02861733 in *CRTC2*.

We visualise four examples to better show the meaning of a high or low MMAD absolute difference together with a high or low minimum ICC. Firstly, in the optimal scenario, the MMAD error is low and ICC is high, demonstrating maximum stability. However, as seen in the example of a *KCNQ2* probe cg25356393, this may be related to the relatively small beta range (Figure 5A).

The next example—of a low MMAD and low ICC—confirms that the beta range may be an important factor because, in some instances, it is narrow. The probe in the example, *RER1* cg04246708, gave a very similar result in each instance and may be considered invariant. Such probes are unlikely to be informative biologically or medically, but could hypothetically be used for normalisation and other analytical purposes (Figure 5B).

When the MMAD increased, even a high ICC did not guarantee that the probe would provide consistent results through time, which is shown in the next example—of the high MMAD and high ICC probe cg11144103 in *PTRF*. There was reasonable agreement, but large absolute errors also occurred (Figure 5C).

Finally, with a high MMAD and low ICC, the probe is not predisposed to offer any stability in time, as with cg02861733 from *CRTC2* (Figure 5D).

**Figure 6:**
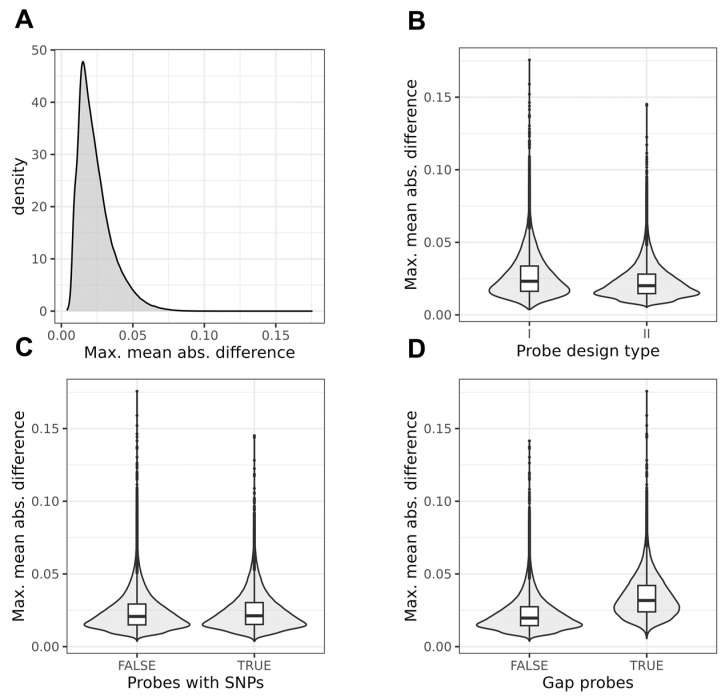
The stability of blood DNA beta methylation values at microarray probes as measured using the maximal mean absolute difference (MMAD) between two timepoints in three cohorts. (**A**) Distribution of values for all analysed probes. For majority of these probes, MMAD was smaller than 0.05. (**B**) Impact of probe design type on MMAD value. (**C**) MMAD values for probes with SNPs in their vicinity. (**D**) Relationship between probes identified by Gaphunter [27] as potentially influenced by genetic variance.

**Figure 7:**
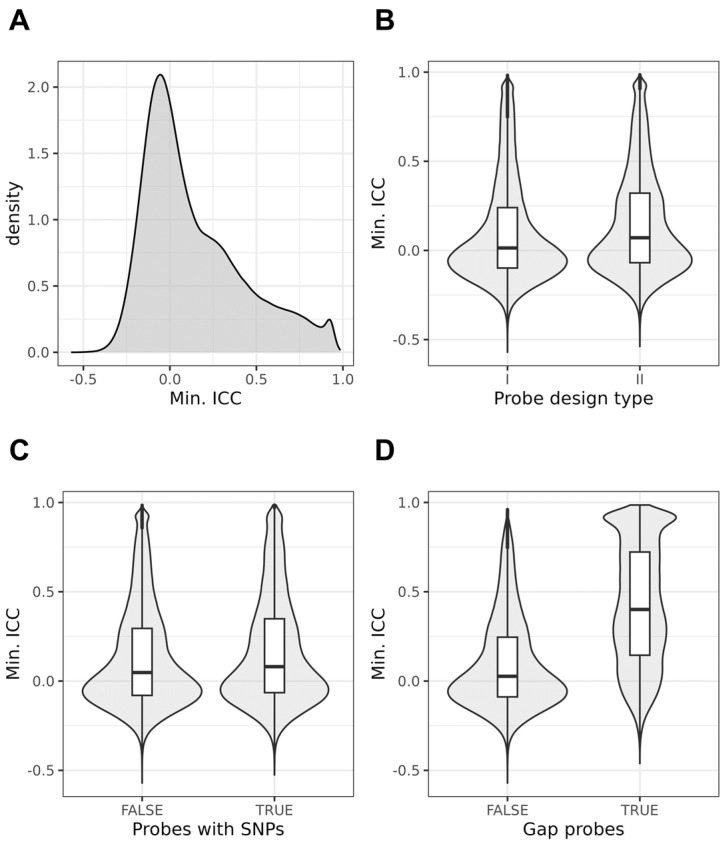
Stability of DNA methylation (beta values) at all analysed microarray probes, determined using the minimal of three intraclass correlation coefficients (ICC) calculated for two timepoints in each of the three datasets. Minimal ICC values (**A**) distribution, (**B**) values depending on probe design type, (**C**) proximity of single-nucleotide polymorphisms (SNPs), and (**D**) potential influence of genetic variance on the methylation level as identified by Gaphunter [27].

Interestingly, no large differences were found when probes with SNPs were compared with the other probes. This is illustrated in Figure 6. Likewise, no strong differences were found for the minimum ICC (from three datasets) (Figure 7).

### 3.6. Hyperstable Probes

**Figure 8:**
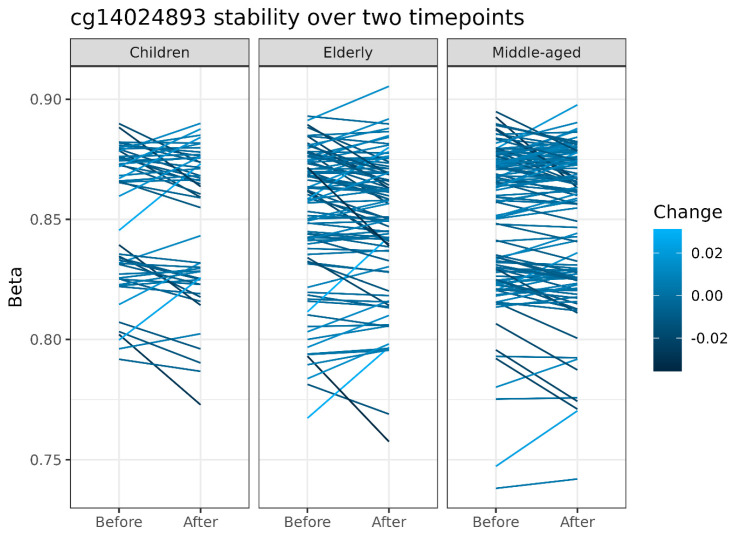
The stability of probe cg14024893 (in the gene encoding ectonucleoside triphosphate diphosphohydrolase 2—*ENTPD2*) between two timepoints for three groups: (1) children, (2) elderly, and (3) middle-aged adults. Although the probe was not identified by Gaphunter [27] as related to genetic variation, two discreet groups can be discerned in children and the middle-aged.

Above, we have inspected the results relative to genetics and probe types, and also reviewed the distributions of the MMAD and the ICC, and their relationships. In the following subsection, we intend to define a set of methylation probes which possess a set of characteristics: (i) independence of genetics, (ii) low MMAD, and (iii) high ICC (iv) across the three analysed datasets. In brief, this section focuses on probes that are the most stable, but do not directly reflect known effects of genetics. We take probes below the 5th percentile of MMAD (<0.010) and above the 95th percentile of the minimum ICC in the three datasets (>0.740). Out of 239 probes meeting the MMAD and ICC criterion, 172 have not been identified by Gaphunter [27], nor are located close to a frequent SNP. These probes, together with complementary information, are listed in Supplementary Table S1. The top 5 probes by highest ICC from among 172 are presented in Table 1. Line plots illustrating the changes in beta values of the top probe (cg14024893) over time are included in Figure 8. Of note, the visual inspection of these data may be suggestive of a genetic influence in some cases (incl. Figure 8), even if the Gaphunter [27] results were negative.

### 3.7. Ontology Analysis of Hyperstable Probes

Hyperstable probes without an evident genetic influence (*n* = 172) were not enriched for gene ontology terms. The 172 hyperstable probes belonged to the following gene location groups: the gene body (79), none (50), TSS200 (19), TSS1500 (16), and UTR (8). The largest number of hyperstable probes were found on chromosome X (17 vs. 10,156 probes analysed), followed by chromosome 19 (14 vs. 24,225), 2 (14 vs. 31,964), 16 (11 vs. 20,748), 1 (10 vs. 42,756), 20 (10), and 5 (10 vs. 22,535).

### 3.8. Agreement Score for Methylation Levels Across Time

**Figure 9:**
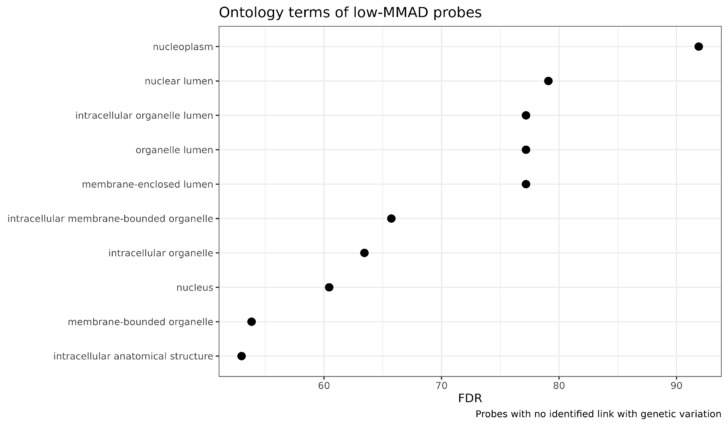
Ontology terms for probes with the lowest MMAD scores. FDR was -log_10_-transformed.

Ontology term enrichment was found for the probes with the lowest MMAD, as shown in Figure 9. They related mostly to organelle terms, and to the nucleus in particular. However, no cellular component (or functional) term enrichment was found for the probes with the highest ICC.

### 3.9. Similarity in Results from 450k EPIC Arrays

The level of correlation of the ICC (all probes) between results from the two studies using 450k arrays (r = 0.780) was similar to that between the 450k and EPIC (850k) arrays (children—middle-aged r = 0.710; and elderly—middle-aged r = 0.784).

With regard to the MMAD, the results from studies using the 450k arrays (r = 0.904) were also similar to those between 450k and EPIC (children—middle-aged r = 0.841; and elderly—middle-aged r = 0.870), suggesting that 450k and EPIC could be analysed together, as in this study.

### 3.10. MMAD and the Level of Methylation and Its Variance

**Figure 10:**
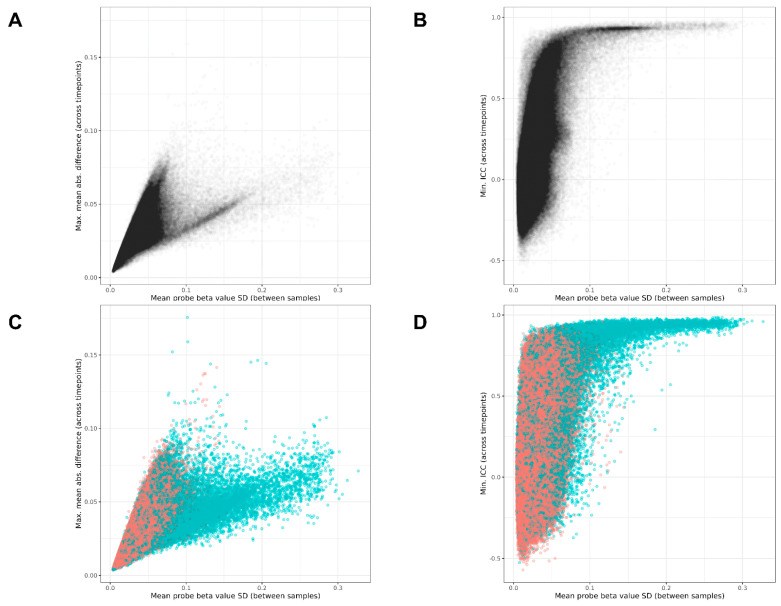
Relationships between MMAD, ICC and the standard deviation of mean beta values. (**A**) Scatterplot of MMAD vs. beta value standard deviation in the datasets: across two timepoints in the same individuals from three cohorts. (**B**) Scatterplot of minimal ICC vs. the beta value standard deviation. (**C**) A modification of panel A with red dots representing probes independent from genetic factors and blue dots indicating probes with relation to genetic factors. (**D**) A modification of panel B with dots in red showing probes unrelated to genetics, and dots in blue indicating probes related to genetics. Probes at which methylation is influenced by genetics are stable in the same individuals on two separate measurements in the blood despite the probes’ overall high between-subject variability (high beta value standard deviation).

A supplementary analysis was carried out to inspect the MMAD relative to the standard deviation (SD) of the beta values, i.e., to find probes that may vary a lot between persons, but which remained at the same level in each person across two timepoints, and this was not attributed to genetics. The SD of the beta values was calculated for each dataset, and a mean for all datasets was obtained (the correlations between SD values in all datasets were r > 0.99). The results for the MMAD (and ICC) are presented in Figure 10A,B. The plots show that many of the lowest MMAD values were achieved in probes with a low population SD (invariant probes). The highest ICC, as could be expected because of its sensitivity to outliers, was found for probes with the highest SD.

The lowest MMAD and the highest ICC were found in probes related to genetic variation (Figure 10C,D). The probes with the lowest proportion of MMAD to SD can be found in the full results, in the Supplementary Table S2, available for download at Zenodo: 10.5281/zenodo.14034923. Additionally, the complete results of the within-group paired comparisons of the methylation levels at two timepoints for all three cohorts are available in Supplementary Table S3.

## 4. Discussion

In this study, we searched for methylation patterns that would be stable across time and independent of genetic variation. To that end, we analysed three datasets generated with Infinium BeadChip arrays, consisting of pairs of samples from pre-/post-pubertal children, elderly people before and after a 10-year follow-up, and middle-aged obese adults before and after an 18-month period of dietary intervention. We detected that the majority of probes were characterised by the considerable variation in methylation levels over time, although there were also probes which proved to be much more stable than the rest. Probes at which the methylation level was affected by genetic factors had the higher stability compared with probes without a detectable genetic influence. On the other hand, a considerable number of relatively stable probes (about a third) remained even after excluding probes impacted by genetics (identified by Gaphunter [27] or located near SNPs). We suspect that many of these probes are, in fact, under the influence of genetic factors, even if not detected by Gaphunter [27]. The hyperstable probes were mostly associated with cell adhesion molecules and ion channels. High variability in most of the probes was an anticipated result. It stands in agreement with previous results and an assumption that the epigenome is heavily influenced by environmental factors. The fast-paced epigenetic adaptation (and/or shifting proportions in cell types) is likely not the only factor underlying the observed high variability, in light of findings that underscore the low reliability of many probes [17]. The higher overall stability in probes influenced by the genotype, as found in the study, was also expected [18,28].

In our study, we focused on detecting the general trends of methylation level stability over long periods of time. This contrasts with the most frequent approach, which is focused on one measurement in time (with or without genetic factor/methylation quantitative trait loci) and the analysis of the link to disease, or the potential for biomarker discovery. Probes under the genetic influence are sometimes removed during analysis, but there are ongoing discussions about whether such an approach is not too stringent, and what the optimal number of probes for exclusion would be [6,28,29,30,31,32]. The results from previous studies, and from this study, suggest that limiting the methylation analysis to a specific set of highly reliable probes could be an adequate strategy. Moreover, if many probes are dependent on the genotype, it may be interesting to check for interactions between the environment and genetic information at these reliable probes.

Apsley et al. [32] analysed the stability of methylation patterns over time (hours) and in the presence of acute psychosocial stress. They showed that stress had a stabilising influence on a subset of probes on relatively longer time intervals. They also analysed probes used in most epigenetic clocks, which had an average or below average stability (a problem also raised in [17]). Their sample size was, however, 31 and they focused on stability in very short periods of time. Talens et al. [29] investigated the variation and long-term stability of methylation patterns, but did so for a few select loci. This sufficed to highlight an important detail—methylation varied considerably in most probes, but was more stable for fully methylated or unmethylated sites. Our research confirms these findings, but with regard to MMAD, and not ICC.

**Figure 11:**
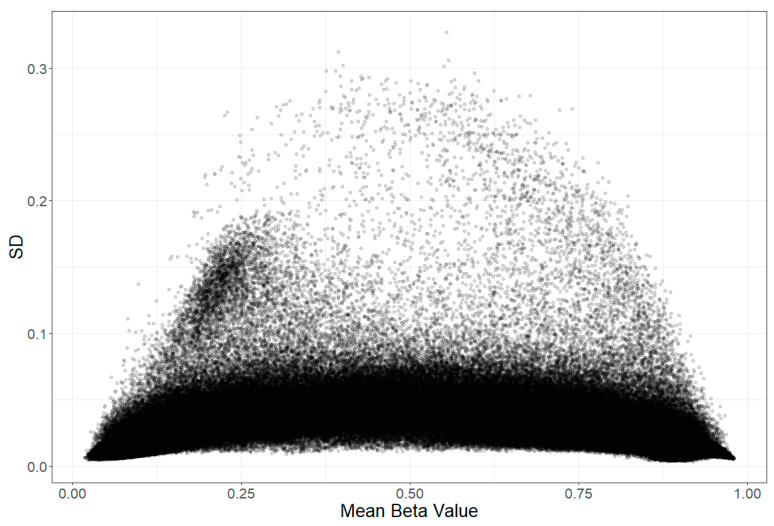
Relationship between the standard deviation (variability) of methylation at analysed probes between samples and their mean methylation level (beta value). For wholly methylated and unmethylated probes, the variation is the lowest. The cluster visible on the left was not enriched for ontology terms but 98.5% of its probes was related to genetic variation. This is unsurprising given that 95.3% of probes with SD > 0.1 were related to genetics as well (compared with 26.6% for probes with SD < 0.1).

Forest et al. compared the stability of methylation levels between repeated runs for the same sample (technical variation), and between samples taken at different times or from different tissues (biological variation). They noted that probes with a high variation across samples (high SD) usually had a high ICC, but also that probes with a low variation across samples did not vary significantly in their differences in methylation levels, and that those probes were either fully methylated or unmethylated. They defined those later probes as “invariable” and focused instead on probes with mean methylation levels within the interquartile range or those with a large inter-individual SD (this overlaps, as shown in Figure 11) [33]. A different approach was taken by Sugden et al. [17], because invariance is a cause of low ICC per se.

**Figure 12:**
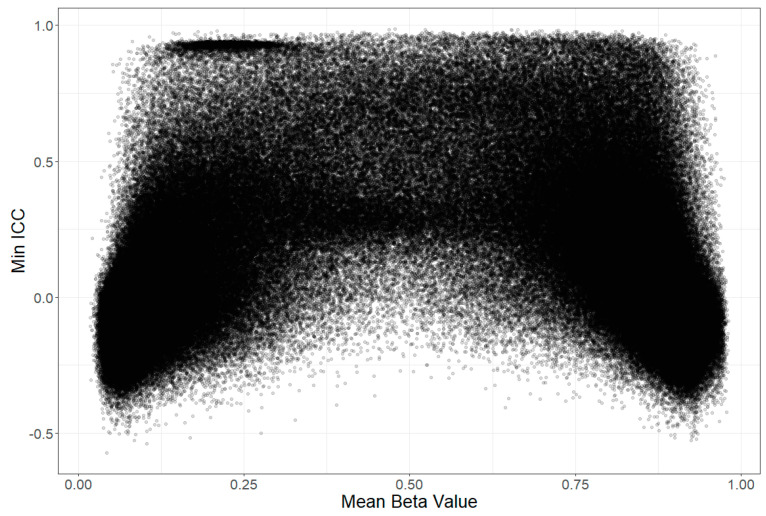
Relationship between the minimal ICC (beta value stability between two measurements in whole-blood DNA) and the mean beta value for all analysed probes. Negative values of ICC can be interpreted similarly to the Pearson’s r coefficient. The horizontal cluster visible in the upper left part of the scatterplot (unmethylated in the most stable manner; min. ICC > 0.74 and mean beta < 0.4) contained almost uniquely probes dependent on genetic variation (97.3%) and was enriched for peptide antigen binding (FDR = 0.000781), luminal side of endoplasmic reticulum membrane (FDR = 0.00923), and the major histocompatibility complex (MHC; FDR = 0.0157).

In accordance with previous findings, where the ICC was used as a measure of probe stability, we define one of our criteria for hyperstability as high ICC (>0.74). While Forest et al. [33] decided to search for stability just in probes that show variance across individuals, we were interested in all stable probes that do not show the influence of the genetic factor; thus, our second criterion was low MMAD. We chose MMAD over SD because, as discussed earlier and also mentioned by Forest et al. [33], the ICC is the greatest when SD is the highest, due to the susceptibility to outliers. As already mentioned, the ICC being close to 0 may be caused by a lack of variability across samples. For that reason, ICC is not a good indicator for probe stability with very high or very low methylation levels [33,34]. Using SD as a criterion would result either in the loss of a substantial portion of probes that have a high ICC or in the exclusion of probes that have a high variability (Figure 11 and Figure 12), either of which can be stable in time [35]. Whether such probes can be considered trustworthy or informative is debatable. Additionally, the majority of probes with a high SD are influenced by the genotype (Figure 10C,D).

**Figure 13:**
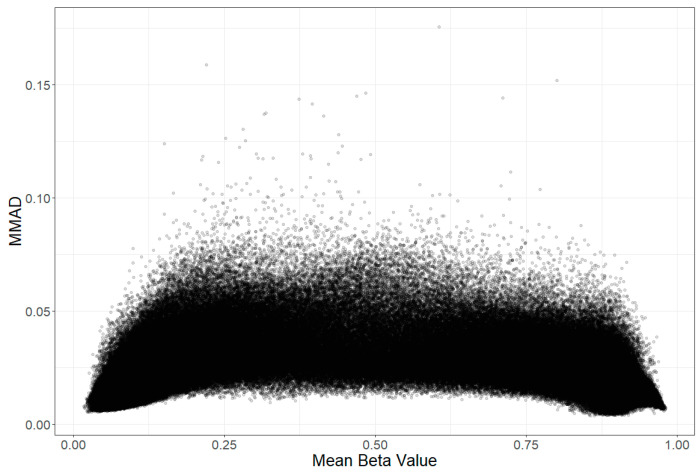
Relationship between MMAD score (beta value stability; may be interpreted as the absolute error) and mean beta values for all analysed probes. Probes with lowest MMAD were found with the lowest and with the highest mean methylation (beta value) across datasets.

As shown in Figure 5C, the ICC alone is not a satisfying unit of stability measurement; it does not guarantee the consistency of results even when at a high value. The concordance correlation coefficient (CCC) may also define stability too liberally. Therefore, a second parameter for stability, like MMAD, is necessary. In contrast to SD, the MMAD does not cut off probes with methylation levels close to 0 or 100, and, depending on the cut-off threshold, still allows probes with a high variation across individuals (Figure 13). Including probes with a high variation is a good argument for raising the MMAD cut-off value, but, considering that the majority of them are influenced by the genetic factor (Figure 10C,D), it does not seem necessary.

The choice of MMAD and ICC for analyses in this study is also subject to another limitation, different from those listed above. The standard deviation was not assessed relative to the mean beta value at a given probe, as would be done by the coefficient of variation or other repeatability/reliability metrics. Such an analysis was precluded by the range of data (0–1) and heteroscedasticity introduced by the logit transformation towards the extremes of this range. Overall, the MMAD served to find the smallest of the differences measured in beta, without consideration for the mean methylation level, and the ICC was employed to demonstrate agreement between measurements after and before the time period. Such a definition of stability is rooted in the presentation of the data as beta values and is less suitable for most extreme beta values, and might also not reflect the relative variability of probes with low methylation.

As stated in the beginning, the populations in the datasets in this research are quite heterogenous regarding age, both between themselves and inside. This allows us to generalise that the detected hyperstable probes remain stable for the majority of a person’s life. On the other hand, the datasets are homogenous with respect to race and ethnicity; thus, the obtained results can only be safely generalised in people of European descent. Prospective future studies should, therefore, include more diverse populations, so that it could be ascertained whether the stability of selected probes persists across all humans. Similarly, it could provide an interesting insight to research what probes remain stable in other tissues, and whether those probes overlap with the main results of this study. Of note, tissues contain cell types in variable proportions, which has an important influence on the overall methylome. Although this research does not provide new packages or functions, it supports the research community with a summary of the stability metrics (Supplemental Data) for individual probes. The invariant probes attract attention as having the potential for strengthening the normalisation methods and verifying the research data integrity, and also as an interesting topic for further research using whole-genome bisulfite sequencing.

Probes with low ICC values are generally unstable over the investigated time period, and it is difficult to draw conclusions about the nature of their epigenetic control. Their instability may reflect the greater responsiveness to minute changes in the current state of the organism and the environment. On the other hand, probes with a low ICC are very plentiful, and, thus, they are quite challenging to interpret, and there is a possibility that the observed effects are stochastic.

Among the hyperstable probes, the one with the highest ICC was cg14024893, which is located in the *ENTPD2* gene. It encodes Ectonucleoside Triphosphate Diphosphohydrolase 2, which is a membrane protein. Studies report using the expression levels of *ENTPD2* for survival prognostication in gastric cancer, lung adenocarcinoma, and COVID-19 [36,37,38]. Two groups analysed the functions of *ENTPD2* in mice with dextran sulphate sodium-induced colitis, and showed that it plays an important role in barrier function, gut motility, and neuromuscular communication [39,40]. Others explored its functions in colon cancer and hepatocellular carcinoma [41,42]. Based on those findings, as well as the exceptional stability of cg14024893, we suspect that using it as a biomarker would offer specific advantages: if this site retains the same level of methylation over the years, a proof would be needed to show that it may change in response to specific conditions.

It is noteworthy that a probe located in the *BRCA1* gene, cg24900425, was also found to be hyperstable. *BRCA1 DNA Repair Associated* (*BRCA1*) is well-known in the field of oncology as it encodes a phosphoprotein which, among other things, acts as a tumour suppressor. As reported in many studies, *BRCA1* mutations are responsible for a large increase in the lifetime risk of breast and ovarian cancers [43], and plays a role in targeting prophylactic interventions and treatment [44,45]. Studies of the *BRCA1* methylation profile suggested its use as an epigenetic biomarker [46]. Considering the hyperstability of cg24900425, we believe that further research into its potential use in diagnostics is warranted.

Hyperstable probes cg17512474 and cg17378342 are both located in the *CDX1* gene, which encodes a transcription factor that is highly specific for the gastrointestinal tract. As its expression is very low in other tissues, including the whole blood, the high stability of cg17512474 and cg17378342 methylation may not have practical relevance. Studies report that the overexpression of this gene hints at a favourable prognosis in gastric cancer cases, and that its methylation patterns may be used as a biomarker in colorectal cancer [47,48]. We therefore suggest that it may be beneficial to study this gene’s and probe’s stability in the tissues of the intestinal tract.

Another probe, cg24342013, is located in the *RB1* gene, which encodes another rather well-known tumour suppressor. The defects in this gene may result in the development of retinoblastoma [49], and many other types of cancer. Moreover, the *RB1* expression level is indicative of adverse outcomes in cancer patients [50,51], which highlights a potential for clinical applications. This extends to *RB1* epigenomics, as methylation patterns in RB1 have been suggested to predict the recurrence of malignant breast tumours [52]. Overall, some of the probes that were found to be hyperstable are located in clinically relevant genes that are under active research focusing on clinical applications, including biomarkers.

## 5. Conclusions

The methylation at most probes that make up the human blood methylation profile was not found to be highly stable. Probes at which methylation is related to the genetic variation appear relatively more stable, but genetics may not be the only factor driving stability, which hints at long-term epigenetic programming. ICC informs us about one facet of methylation stability over time and other metrics should be taken into consideration as well, such as the absolute difference (error). The value of invariant probes is uncertain, but their stability might provide new opportunities of data analysis. A resource presenting the stability of blood DNA methylation at 450k probes over time is made publicly available, and might be used in conjunction with studies specifically focused on reliability [17]. Further research in the area is warranted, embedded in long-term studies, to find out if the stability of probes differs depending on health or disease in different tissues and populations.

## Figures and Tables

**Table 1:** Example of top 5 probes by highest ICC from among most stable probes (with the lowest MAD, and highest ICC) with no SNP or gap identified by Gaphunter [27]. The *p*-value from the paired t-test is included. All the 172 probes were of design type I. Interestingly, *WDR27* is thought to be an imprinted gene.

Probe	cg14024893	cg27404186	cg08084154	cg09894276	cg27388297
Chromosome	9	2	16	6	X
Coordinate	139943146	242843821	2848577	169977394	52897160
Gene	*ENTPD2*	*(FAM240C)*	*TESSP1*	*WDR27*	*XAGE3*
RefGene Group	Body	None	1stExon	Body	TSS200
**Max. MAD from 3 age groups**	**0.00941**	**0.00689**	**0.00942**	**0.00851**	**0.00892**
MAD—children (450k)	0.00941	0.00689	0.00825	0.00851	0.00874
MAD—elderly (450k)	0.00841	0.00676	0.00916	0.00831	0.00892
MAD—middle-aged (EPIC)	0.00689	0.00647	0.00942	0.00696	0.00674
Max. MAD/SD	0.314	0.314	0.299	0.305	0.345
Mean SD from 3 age groups	0.0299	0.0219	0.0314	0.0278	0.0258
**Min. ICC from 3 age groups**	**0.917**	**0.915**	**0.913**	**0.906**	**0.897**
ICC—children (95%CI)	0.917 (0.86–0.952)	0.916 (0.858–0.951)	0.95 (0.914–0.971)	0.915 (0.857–0.951)	0.919 (0.862–0.953)
ICC—elderly (95%CI)	0.923 (0.885–0.948)	0.916 (0.876–0.944)	0.915 (0.874–0.943)	0.906 (0.861–0.937)	0.898 (0.849–0.932)
ICC—middle-aged (95%CI)	0.958 (0.941–0.971)	0.927 (0.896–0.949)	0.914 (0.879–0.939)	0.945 (0.922–0.961)	0.941 (0.917–0.959)
**Min. paired *t*-test p from** **3 age groups**	**0.129**	**0.0701**	**0.176**	**0.457**	**0.323**
Paired *t*-test p—children	0.214	0.270	0.176	0.457	0.323
Paired *t*-test p—elderly	0.129	0.208	0.724	0.643	0.894
Paired t-test p—middle-aged	0.260	0.0701	0.350	0.492	0.774
Probe design type	I	I	I	I	I

450k—the Infinium HumanMethylation450 array; EPIC—the Infinium MethylationEPIC array; ICC—intraclass correlation coefficient d(agreement, two-way); MAD—mean absolute difference; Max.—maximum; Min.—minimum; SD—standard deviation of beta values (specifically: the mean of the three means of standard deviation of beta values across the three datasets/age groups).

## Data Availability

The original data are publicly available (details are provided in the Materials and Methods section).

## Supplementary Materials

The following supporting information can be downloaded at: https://www.mdpi.com/article/10.3390/biomedicines12112557/s1, The link to full results (external repository) will be added at the latest stage. Full results can be found in Supplementary Table S2, available for download at Zenodo: 10.5281/zenodo.14034923.
